# Sodium-glucose Cotransporter 2 Inhibitors and Ketoacidosis – Clinical Implications in the Treatment of Patients with Type 2 Diabetes

**DOI:** 10.17925/EE.2016.12.01.33

**Published:** 2016-03-15

**Authors:** Kirsten Stinkens, Chantal Mathieu

**Affiliations:** Department of Endocrinology UZ Leuven, UZ Gasthuisberg, Leuven, Belgium

**Keywords:** Sodium-glucose cotransporter 2 inhibitors, ketoacidosis, type 2 diabetes

## Abstract

The use of sodium-glucose cotransporter 2 inhibitors is associated with an increased risk of diabetic ketoacidosis. This risk has been reported in particular in off-label use of these agents in type 1 diabetes, but reports of risks in type 2 diabetes patients also exist. In type 2 diabetes ketoacidosis is rare and is present particularly in patients who have undergone prolonged starvation, serious infection, alcohol abuse or surgery. The pleiotropic advantages of sodium-glucose cotransporter 2 inhibitors do not outweigh the risk for a diabetic ketoacidosis, but caution is warranted.

Sodium-glucose cotransporter 2 inhibitors (SGLT2i) are the latest class of oral glucose-lowering agents proposed for the treatment of patients with type 2 diabetes mellitus (DM2). Besides lowering blood glucose in an insulin-independent manner by blocking the tubular reabsorption of filtered glucose, they also have a positive effect on blood pressure, weight control and albuminuria, making them true anti-diabetic agents.^1–5^ In addition, they have the advantage of not causing hypoglycaemia and, recently, the Empagliflozin, Cardiovascular Outcomes, and Mortality in Type 2 Diabetes (EMPA-REG OUTCOME) trial showed a relative reduction in cardiovascular mortality of 38% compared with placebo.^6^ These beneficial effects have rapidly rendered these agents drugs of choice for treatment of patients with DM2. However, recent reports on the occurrence of ketoacidosis with these agents have led the regulatory agencies (Food and Drug Administration [FDA] and European Medicines Agency [EMA]) to issue a warning on the matter of SGLT2i.^7,8^ Should clinicians worry?

The worry is mainly based on interesting case reports, as the EMPA-REG Outcome trial did not show a signal for ketoacidosis. The largest patient group was described by Peters et al., with 13 cases of diabetic ketoacidosis (DKA) in a total of nine patients being reported. However, only two of them had DM2, which is the only official indication for prescribing an SGLT2i. These two patients developed DKA 12 hours and one week after surgery, respectively. Six out of seven patients with type 1 diabetes mellitus (DM1) had reduced their insulin dose before the episode of DKA. Four cases had an associated respiratory or gastrointestinal infection and three used alcohol previous to the DKA.^9^ Researchers at Janssen Pharmaceuticals also published an article about all serious adverse events of DKA and related events (ketoacidosis, metabolic acidosis, and acidosis) from 17,596 patients from randomised studies of canagliflozin.^10^ In total, 12 patients developed a DKA, with only 10 of them being treated with SGLT2i. In addition, six out of 10 patients were shown retrospectively to have glutamic acid decarboxylase (GAD) autoantibodies, suggestive of autoimmunity, indicating that they were not typical DM2 patients. Of interest, also in these patients there was often an eliciting event like surgery, alcohol or an infection.

When evaluating the available case reports described in the literature it is striking that most cases occurred in patients with severe betacell dysfunction or with undetected autoimmune diabetes. In addition there was often a trigger like alcohol abuse, an infection and prolonged starvation, as with surgery. Another important point is that the clinical diagnosis of DKA was not always convincing because in many cases there were missing pH values and, often, no measurements of urinary and/or blood ketones.

Patients treated with SGLT2i, however, may have elevated levels of ketones, without it being pathological. SGLT2i lower blood glucose by inducing glucosuria in an insulin-dependent manner; therefore, the pre- and postprandial blood glucose levels will be lower. Patients who are treated with insulin will have to reduce their insulin dose in order to prevent hypoglycaemia – also the endogenous insulin production will be lower. The insulin dose can come below a critical point that is necessary to inhibit lipolysis and prevent ketogenesis.^11^ Recently, a direct effect of SGLT2i on the alpha-cells of the islets of Langerhans has also been described. The production of glucagon will increase, causing a decline in insulin:glucagon ratio with stimulation of lipolysis, gluconeogenesis and ketogenesis.^12^ Animal studies also show that SGLT2i will lead to a rise in reabsorption of ketone bodies.^13^ In conclusion, patients treated with SGLT2i will have less insulin and more ketones in basal circumstances. However, when these patients reduce their carbohydrate intake, in order to lose more weight, encouraged by the already favourable weight effect of SGLT2i, they may further have to reduce their insulin dose, leading to even more lipolysis and ketogenesis. Furthermore, elevation of the counter-regulating hormones (cortisol, glucagon, adrenaline, growth hormone) such as in severe stress (i.e. surgery of infection) will stimulate peripheral lipolysis and lead to the formation of ketones. Eventually the accumulation of ketones may lead to a DKA.

Based on our current knowledge of SGLT2i and the described case reports it can be concluded that the risk of DKA when taking a SGLT2i is very low in patients with DM2 and is especially seen in patients with low or absent beta-cell function. Prolonged starvation, serious infection, alcohol abuse or surgery can predispose a patient to DKA. In these conditions increased awareness and even temporary discontinuation of the drug is advised, with the average half-life of most SGLTi being 12 hours.^14^ Finally, clinicians have to be aware of two major clinical pitfalls. First, due to the insulin-independent-induced glucosuria, the glycaemia may not be profoundly elevated when the patient presents with a DKA. Second, urinary ketones can be elevated in patients taking SGLTi without being pathological, but urinary ketonelevels may also be deceivingly lowered by the SGLT2i, making it crucial to check blood ketones (beta-hydroxybutyrate) rather than urinary ketones when DKA is suspected in a patient taking SGLT2i.

## References

[1] Monami M., Nardini C., Mannuci E. (2014). Efficacy and safety of sodium glucose co-transport-2 inhibitors in type 2 diabetes: a meta-analysis of randomized clinical trials. Diabetes Obes Metab.

[2] Yang X. P., Lai D., Zhong X. Y. (2014). Efficacy and safety of canagliflozin in subjects with type 2 diabetes: systematic review and meta-analysis. Eur J Clin Pharmacol.

[3] Liakos A., Karagiannis T., Athanasiadou E. (2014). Efficacy and safety of empagliflozin for type 2 diabetes: a systematic review and meta-analysis. Diabetes Obes Metab.

[4] Fioretto P., Giaccari A., Sesti G. (2015). Efficacy and safety of dapagliflozin, a sodium glucose cotransporter 2 (SGLT2) inhibitor, in diabetes mellitus. Cardiovasc Diabetol.

[5] Stanton C. (2014). Sodium glucose transport 2 inhibition decrease glomerular hyperfiltration. Circulation.

[6] Zinman B., Wanner C., Lachin J. M. (2015). Empagliflozin, cardiovascular outcomes and mortality in type 2 diabetes. N Engl J Med.

[7] US Food and Drug Administration Drug Safety Communication: FDA warns that SGLT2 inhibitors for diabetes may result in a serious condition of too much acid in the blood. http://www.fda.gov/safety/medwatch/safetyinformation/safetyalertsforhumanmedicalproducts/ucm446994.htm.

[8] European Medicines Agency (2015). Review of diabetes medicines called SGLT2 inhibitors started: risk of diabetic ketoacidosis to be examined. http://www.ema.europa.eu/ema/index.jsp?curl=pages/medicines/human/referrals/SGLT2_inhibitors/human_referral_prac_000052.jsp&mid=wC0b01ac05805c516f.

[9] Peters A., Buschur E., Buse J. (2015). Euglycemic diabetic ketoacidosis: a potential complication of treatment with sodium-glucose cotranspoter 2 inhibition. Diabetes Care.

[10] Erondu N., Desai M., ways K. (2015). Diabetic ketoacidosis and related events in the Canagliflozin Type 2 Diabetes clinical program. Diabetes Care.

[11] Ferrannini E., Muscelli E., Frascerra S. (2014). Metabolic response to sodium-glucose cotransporter 2 inhibition in type 2 diabetic patients. J Clin Invest.

[12] Bonner C., Kerr-Conte J., Gmyr V. (2015). Inhibition of the glucose transporter SGLT2 with dapagliflozin in pancreatic alpha cells triggers glucagon secretion. Nat Med.

[13] Cohen J. J., Berglund F., Lotspeich W. D. (1956). Renal tubular reabsorption of acetoacetate, inorganic sulfate and inorganic phosphate in the dog as affected by glucose and phlorizin. Am J Physiol.

[14] Scheen A. (2015). Pharmacodynamics, Efficacy and safety of sodium-glucose co-transporter type 2 (SGLT2) inhibitors for the treatment of type 2 diabetes mellitus. Drugs.

